# On the Spontaneous Build-Up of Voltage between Dissimilar Metals Under High Relative Humidity Conditions

**DOI:** 10.1038/s41598-020-64409-2

**Published:** 2020-05-06

**Authors:** J. Y. Lax, C. Price, H. Saaroni

**Affiliations:** Porter School of the Environment and Earth Sciences, Faculty of Exact Sciences, Tel Aviv University, Tel Aviv, Israel

**Keywords:** Environmental sciences, Materials science, Atmospheric science

## Abstract

Certain metals can surprisingly build-up charge spontaneously, when exposed to high relative humidity (RH), although they need to be isolated from the ground. We have explored this phenomenon, building on former experimental knowledge and carrying out additional experiments, to identify the parameters that could enhance this charging. We used many types of metals with different characteristics under different RH and temperature conditions. While some metals were unaffected by high RH, others, like zinc and stainless steel, did acquire charge, when RH was >60%, and charged a capacitor to a voltage of 1 V. For the first time, we also performed outdoors experiments, showing this phenomenon is also valid under similar natural ambient humid conditions. If these results can be scaled up, it may lead to the development of practical applications for regions and times of high RH conditions.

## Introduction

Since the days of Benjamin Franklin (1752), we know that the atmosphere and thunderstorms are electrified. However, even today we still do not fully understand these phenomena^1^, although we do know that the key ingredient is the interaction between water molecules in their different phases (vapor, liquid, and ice)^1–3^.

Pure water has zero net electric charge, according to the electro-neutrality principle^4^. There are positive ions (H+) and negative ions (OH-) but the number of these ions balance each other resulting in electric neutrality. However, as in thunderstorms, water and ice can transfer some of these ions to other particles during melting, freezing and collisions^5^.

In 1843 the famous physicist Michael Faraday published a research related to a powerful electrocution of a worker next to a steam boiler in London^6^. This phenomenon was discovered by Lord William Armstrong and was later known as ‘The Armstrong Effect’^7^. The escaping steam somehow electrified the metal boiler giving the worker a severe shock^6^. Faraday carried out experiments and concluded that the electricity was due to the friction of the water droplets in the steam with the surrounding metal. When no condensed water was present in the steam path, the metal was not electrified. Having enough condensed water droplets in the steam immediately generated electricity. By increasing the pressure of the steam Faraday could increase the effect. He concluded that the effect was due to the condensed water becoming a good conductor, with the ions being transferred to the metal, or any other body, by friction. Faraday also measured the charge on the objects, and concluded that the friction of water droplets against many different bodies left the water positively charged relative to the solid bodies.

Since then, numerous studies regarding the ability of water to become charged by friction^8^, phase change^3,9^, contact electrification^10–13^ and other mechanisms were conducted^14^. Some even tried to harness the charge separation generated by water on surfaces in order to develop a new source of energy^11,15,16^.

It is known that surfaces tend to lose their electrostatic charge when the air is humid since it neutralizes the surface^17^: Under high enough relative humidity (RH > 60%), water molecules will be adsorbed and the surface will be covered with a thin film of water^18^, allowing mobile ions to move and dissipate the accumulated charge. The changed electrical properties, due to the cover of water film as a function of the RH level and surface wetting, is also a major field of interest, and the formation of water upon surfaces, both metals and non-conducting materials, hydrophobic and hydrophilic is vastly studied^19–21^. However, surprisingly, surfaces adsorbing water can, in some cases, gain charge from the humid atmosphere^22^.

Experiments of a recent study^23^ have shown that some metals can acquire spontaneous charge build-up when exposed to high RH conditions (>50%). Different metals charge with different polarity, due to the selective adsorption of water ions (OH− & H+), according to the acid-base characteristics of the metal surface. Aluminum and chrome-plated brass (CPB) became negatively charged, while Stainless Steel (SS) and NiCr (nichrome) became positive. However, some metals, like copper, accumulated negligible charge even under 95% RH. They also found that using two different metals exposed to high RH leads to voltage accumulation, acting as a capacitor, reaching 0.75 V.

A follow up paper tried to determine the acid-base characteristics of a solid surface at the nanoscale^24^. A Kelvin Force Microscope was used, and the study verified that depending on the Brønsted acid or base characteristics of the sample surface – it will adsorb more OH- or H+ selectively, as a function of the RH.

In this study we investigate the generation of electricity during the exposure of different metals to high RH conditions. We aim to further explore the voltage accumulation under controlled lab conditions, using additional metals, while creating conditions closer to natural outdoor conditions and, for the first time, deriving these experiments also under ambient outdoor conditions. In Section 2 we present the methodology of the experiments, in Section 3 the results and discussion, and in Section 4 the conclusions.

## Materials and methods

**Figure 1:**
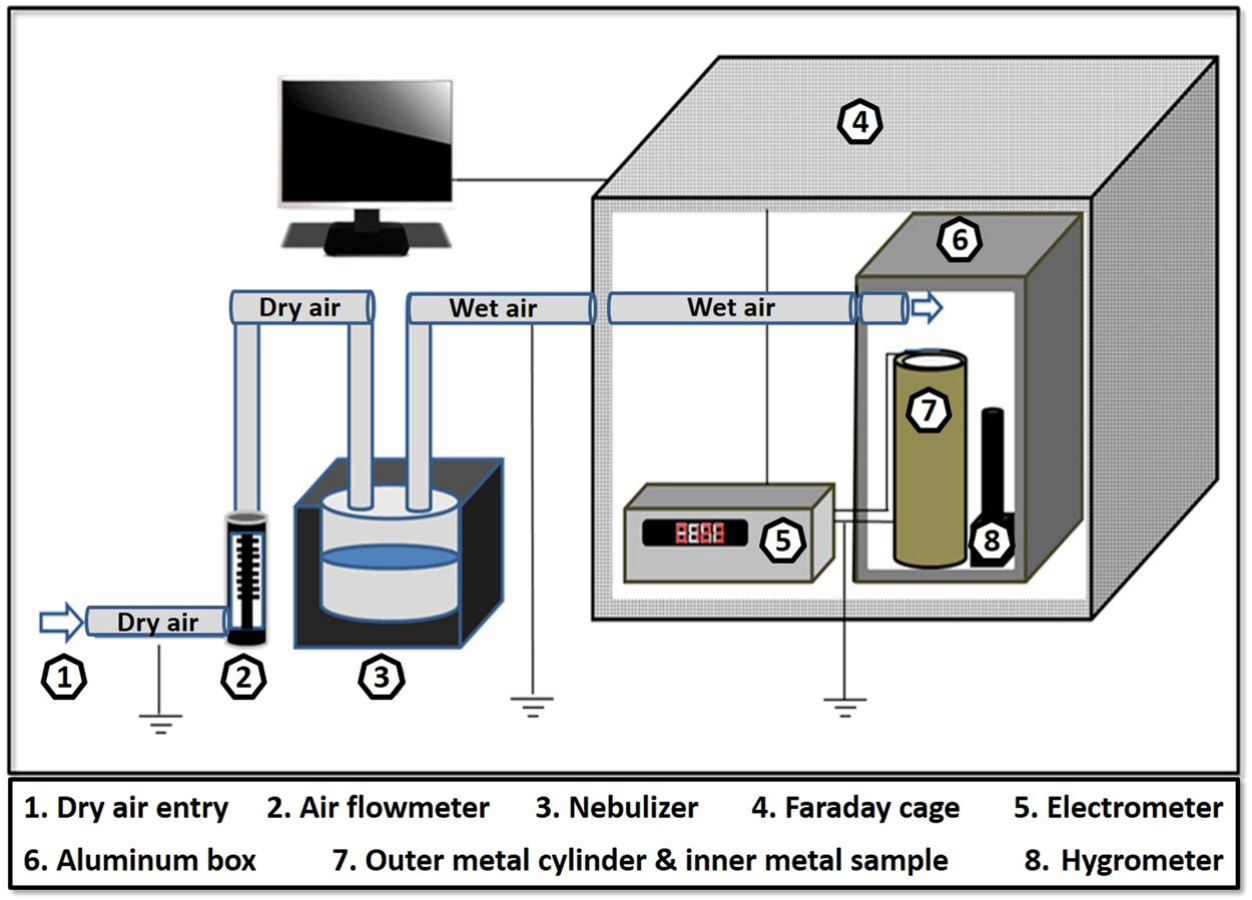
Schematic experiment setup.

The experimental setup was built based on previously published experiments^23^ and includes the following instrumentation: Dry airflow enters a flowmeter for regulation, and then moves into an ultrasonic humidifier (Ultra-Neb99 by DeVilbiss) which injects moisture to the incoming air (Fig. 1). Both dry and wet air pipes are grounded. The wet air enters a closed aluminum box, mounted within a Faraday cage. Inside the aluminum box are mounted an outer chrome plated brass (CPB) cylinder and the inner inspected metal sample. The metals were first grounded, and then voltage was measured between the inspected metal and the surrounding grounded metal (CPB). The inspected metal is connected to a 0.15 μF capacitor on its one end, which is also grounded on its other end. The outer cylindrical metal is grounded, so the voltage measured is accumulated on the capacitor, generated solely from the inner metal with respect to the grounded metal.

Our experiments were divided to three parts. The first set of experiments was executed under constant room temperatures with different RH levels, starting from very dry conditions (RH = 10%) to almost saturated air (95%). The RH and temperature were measured by a hygrometer (Lutron HT-3027SD), with a specified response time of 1 s. Voltage was measured with an electrometer (Keithley 6514), placed within the Faraday cage, able to detect voltages starting from10^−5^ V. Initially we used metals which participated in the former experimet^23^ – (CPB, copper, aluminum, nichrome and stainless steel) – for comparison, but we also explored many other types and shapes of metals (Table 1, Supplementary Table S1) and we also examined how changes in ambient temperature affects the voltage accumulation, all in order to further investigate this phenomenon.

**Table 1:** Voltage measured between an isolated inspected metal and a cylindrical grounded CPB.

Inspected Metal	Shape	Max. Voltage between Metal & Grounded CPB	Total # of Experiments
Stainless Steel (SS) 301, 303, 304, 316, Other	Rod, cable, foil, plate, cuboid, mesh	−0.9 V	56
Zinc (Zn)	Plate, foil, cylinder	−0.9 V	84
Aluminum (Al)	Plate, foil, cylinder	−0.7 V	18
Tin (Sn)	Rod	−0.5 V	3
Indium (In)	Wire	−0.5 V	3
Copper (Cu)	Wire rod, wool	−0.18 V	4
Molybdenum (Mo)	Rod	−0.17 V	5
Nickel (Ni)	Rod	−0.16 V	3
Nichrome (NiCr)	Wire	−0.018 V	2
Silver (Ag)	Plate	−0.015 V	2
Chrome Plated Brass (CPB)	Cylinder	−0.015 V	4
Brass	Plate	−0.015 V	2
Control No metal connected	—	+/−0.02 V	5
Tungsten (W)	Rod	+0.5 V	6
SS wool with Cu wire	Wool & wire	+0.84 V	10

In order to make sure there was no external cause that might give a false reading, we carried out several control experiments in lab to prove that the charge accumulation is indeed generated solely by the high RH. We checked voltage accumulation starting from very dry air, (RH = 10%) up to RH of 95%. We also used different metals (Table 1) as well as no metal at all, in order to see the differences between the cases in terms of voltage accumulation. It should be noted that beyond previous experiments described in the introduction section^23^, our experiments may demonstrate more realistic and natural outdoor conditions. In addition, we checked the importance of the presence of the surrounding Faraday cage on the results, the influence of the source of humidity, the impact of distilled or tap water, and the use of regular air instead of N_2_ as used in Ducati *et al*.^23^.

The second set of experiments took place with different metals and arrangements where asymmetrical capacitors were built using two different metal plates placed one on top of the other with a paper sheet between them to prevent contact. The metals were rolled together and the voltage was measured between them, as the RH increased. Here too, we used a capacitor made of the same metals used in the former experiment (SS mesh & aluminum) for comparison, in addition to other combinations of two dissimilar metals – SS_301_ & SS_316LS_, zinc & aluminum, zinc & SS_316LS_ and SS mesh & SS_316LS_.

While all previous studies conducted experiments under controlled laboratory conditions, our third set of experiments included uncontrolled, outdoor experiments, carried out in the outside ambient air. The same experimental setup was used, excluding the humidifier and the dry airflow. The entire setup was opened in order for the ambient air to freely enter the aluminum box. The experiments took place during several summer days in Tel-Aviv, Israel, located near the coastline, known to exhibit persistent sultry conditions with RH that can exceed 60% during day and 80% during night^25,26^, as well as in Kiryat Ono, Israel, downwind from the city center. For a comparison, measurements were also taken under an extreme hot and relatively dry day that occurred in the last summer^27^. The RH and temperature measured in the experiment chamber were compared with the nearest meteorological station.

## Results and discussion

### Controlled laboratory experiments

**Figure 2:**
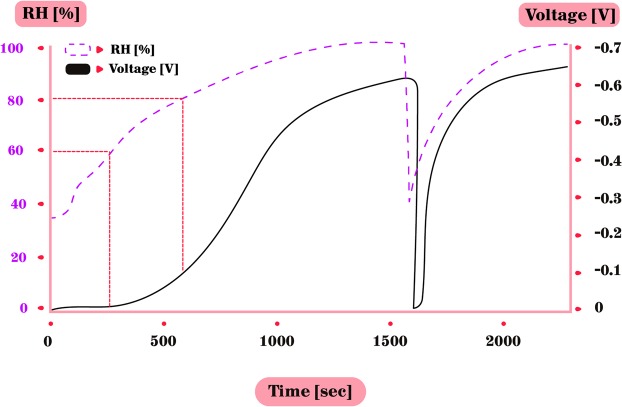
Voltage (V) accumulation between zinc and CPB (black line). The RH (%) is shown in dashed line. V start accumulating when RH >60%. Accumulation rate speeds up at RH >75%. Chamber was then opened to decrease the RH, followed with grounding and re-connecting the metals. The V soon reached same rate as before.

Measurements of electrical potential between the grounded CPB cylinder and numerous different isolated metals have been carried out many times, while changing the RH (Table 1). Not surprisingly, known as an often sacrificial anode in preventing galvanic corrosion due to being more active than most metals^28^, zinc was the most successful in terms of voltage accumulation and reproductively of experiments among the tested metals. Hence zinc was the main focus in the further experiments: As long as the RH was low (<60%), the voltage between the zinc and the grounded cylindrical CPB, remained negligible. However, as the RH passes 60%, voltage starts to accumulate - the higher the RH, the faster the pace of the accumulation (Fig. 2). Note that previous experiments defined the RH threshold of voltage accumulation as 50%^23^. We used several types of zinc – plates, foil and cylinder. In our experiments, the voltage accumulated only from RH of 60%, reaching between −0.65 V and a maximum of −0.9 V without increasing further, even after several hours under high RH. The −0.9 V maximum is not shown in the figures due to the fact that the majority of experiments reached lower voltages.

**Figure 3:**
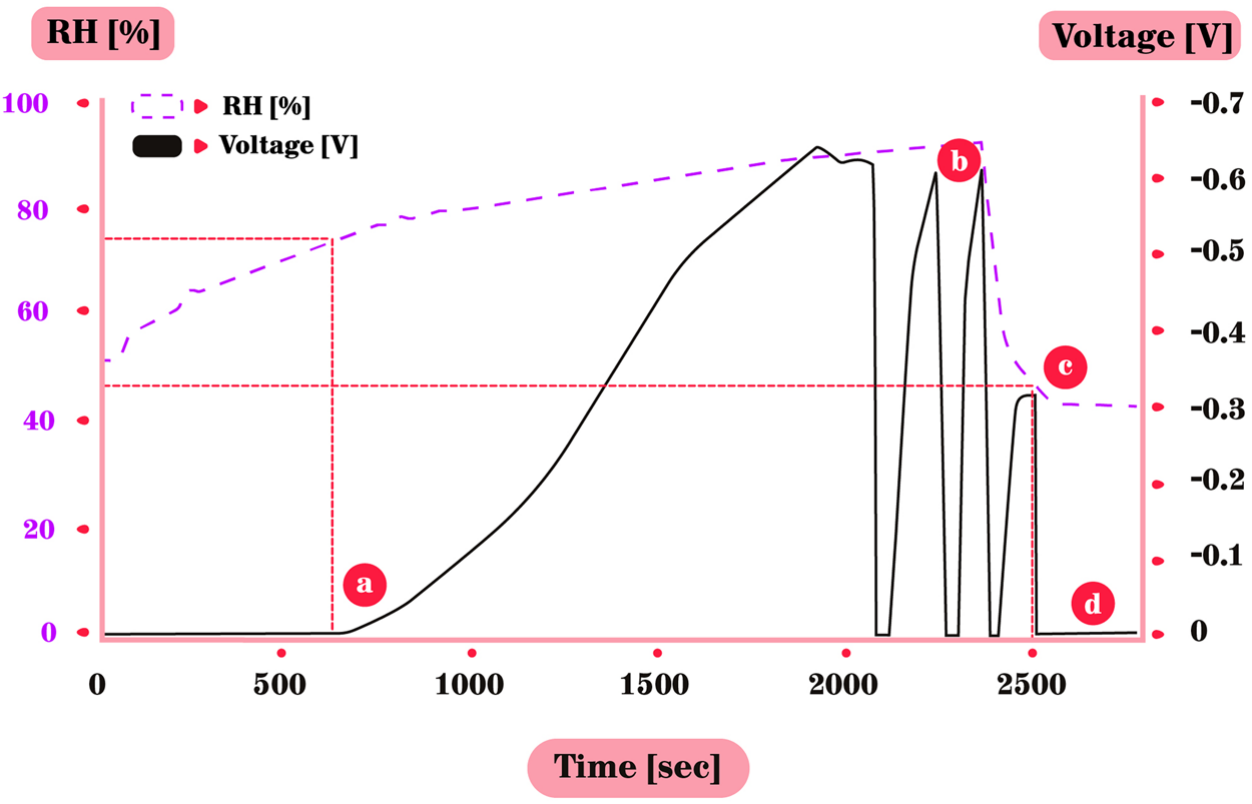
Voltage accumulation duration under different RH levels. (**a**) V accumulation between zinc and grounded CPB only started with high RH. (**b**) Two cycles of grounding & re-connecting metals under high RH. Voltage quickly recovered to former maximum and was then immediately grounded as it reached −0.6 V. (**c**) After grounding and re-connecting, RH was intentionally decreased rapidly by letting ambient air entering the chamber. Voltage reached only half of former maximum, probably due to remaining water on the surface. (**d**) No voltage accumulated after last grounding and re-connecting under low RH (40%).

Furthermore, as long as the RH was higher than 60% it did not lose its accumulated voltage and charge was stored upon the metal. When the RH decreased to be lower than 60%, the voltage started to decrease as well, indicating that the metal is dehydrated and the charged particles dissipate. The first voltage accumulation takes longer than successive groundings and reconnecting of the metals, which was followed by a rapid charging to the former maximum voltage (Fig. 3), in this case −0.65 V. Note that due to the response time of the hygrometer to changes in RH, the actual lag between the rising RH and the rising voltage may be slightly different in reality. This rapid increase of voltage after grounding may indicate that the formation of the thin water film covering the surface, under the high RH, is crucial for the charge built up. In the next 2 cycles of grounding & reconnecting, in order to understand the duration to reach the same voltage under high RH (already present in the chamber), we decided to measure the time it will take to reach a threshold of −0.6 V. These two cycles were grounded the moment the voltage reached −0.6 V – and one can see (in Fig. 3) it takes a very short time for the voltage to accumulate to the prior level compared to the first voltage build up. The importance of high RH in generating this voltage accumulation was also verified by another grounding and reconnecting, while lowering the RH (Fig. 3), which led to decreased voltage accumulation. After another grounding, while RH is low, no voltage accumulation occurred at all.

When no metal was connected to the copper alligator end of the voltage connector, negligible accumulation of voltage was evident under high RH, even after a long period of time.

Next, we investigated the question of whether high RH alone is responsible for the charging of metals, or is the absolute amount of water molecules, i.e., the absolute humidity (AH) important too. Two experiments with similar RH, but under different temperatures were carried out, expressing different AH values. Higher temperatures with high RH indicate larger absolute mass of water molecules per cubic meter compared to the number of water molecules in colder temperatures under the same RH. This is according to the following relation between RH and AH^29^:

$$AH[g/{m}^{3}]=\frac{6.112\cdot {e}^{\left(\frac{17.67\cdot T}{T+243.5}\right)}\cdot RH\cdot 2.1674}{273.15+T}$$

For instance, keeping the RH constant at 80% will indicate an AH of 24.3 [*g*/*m*^3^] with temperature of 30 °C, but an AH of only 7.5 [*g*/*m*^3^] when the temperature is 10 °C. While exploring the duration to reach −0.7 V on a zinc plate, it was noticeable that under the same RH, as the AH increased (with temperature increase), the duration to reach −0.7 V decreased. The higher the AH the shorter the time to reach max V after grounding. It took between 5.5–9.5 minutes to reach max voltage with AH of approximately 23 g/m^3^ (T = 26 °C). However, when the AH was higher than 25 g/m^3^ (28 °C < T < 29.5 °C), the duration decreased to less than 100 seconds. An increase of just 2 °C dropped the charging time to less than a fifth. In a different experiment, temperatures were decreased to 15 °C, so although the RH was high (80–85%), the AH was very low (10.5 g/m^3^) and the time to reach −0.7 V took 29 ± 4 minutes. Either way, the voltage of the zinc reached similar voltages for very different AHs (between 10.5–27.5 g/m^3^), the difference was only in the duration to reach this maximum.

**Figure 4:**
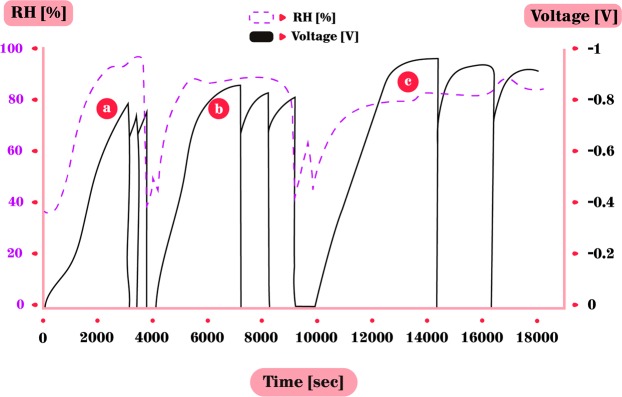
Zinc after dipping in NaCl solution. (**a**) Zinc plate voltage accumulation (max −0.8 V). (**b**) Zinc plate after soaking in salt solution (0.188 M) and drying reached −0.85 V, though decreased with every cycle. (**c**) Zinc plate after soaking in salt solution (0.376 M) and drying reached max −1 V. Once again the Voltage decreases with every grounding-reconnecting cycle.

In order to understand if there is a way to increase the charge accumulation upon the metal, we then decided to increase condensation nucleation sites by first dipping the zinc plate in a NaCl solution (0.19 M). The metal was then dried. Now with salt grains on the zinc plate surface, the voltage reached higher voltages (Fig. 4) vs clean zinc. Doubling the concentration of NaCl in the solution (0.38 M) was followed by an increase in maximum voltage. With every grounding and re-connecting cycle, the maximum voltage decreased slightly, which may imply that the NaCl is gradually being removed from the surface of the metal by the water vapor.

Summarizing the findings from the different metals tested, in the case of silver and nichrome – there was negligible difference in voltage (±0.018 *V*) when increasing RH to as high as 95%, which is similar to the voltage when no inner metal is connected at all (±0.02 *V*). A small voltage accumulation was witnessed by using copper or nickel (−0.018 *V* ± 0.02 *V*). There was almost no difference in voltage between different shapes of copper – wool, wire and rod, though they exhibit some major differences in surface area and roughness. Different behavior was witnessed with other metals, such as zinc, aluminum, several types of SS, indium, tin and tungsten – all measurements showed increasing voltage as the RH crossed a certain threshold. However, here, as well, using zinc in different shapes – foil, plate and cylinder, generally reached the same voltages. Voltages between most of the metals we tested and the grounded CPB were negative, however tungsten or a SS wool with a copper wire as its backbone, reached positive voltages. A summary table of metals and the accumulated voltages is presented in Table 1. Compared to prior experiments showing no charge accumulation for copper and brass – we also received negligible readings of voltage for brass and only small voltages for copper. On the other hand, prior experiments showed charge accumulated on SS, aluminum, CPB and NiCr. We failed to achieve any voltage accumulation upon CPB or NiCr, which might be due to differences between the components of these metals; hence we might not use the exact same materials. We did, however, successfully measure voltages increasing on SS and aluminum and other types of metals as shown in Table 1.

### Asymmetrical capacitors

**Figure 5:**
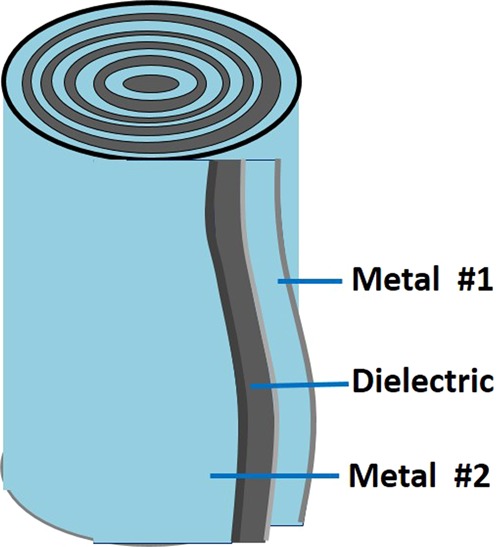
Asymmetrical capacitors made of two dissimilar metals with a dielectric sheet between them. The capacitor is exposed to humidity from all directions.

**Figure 6:**
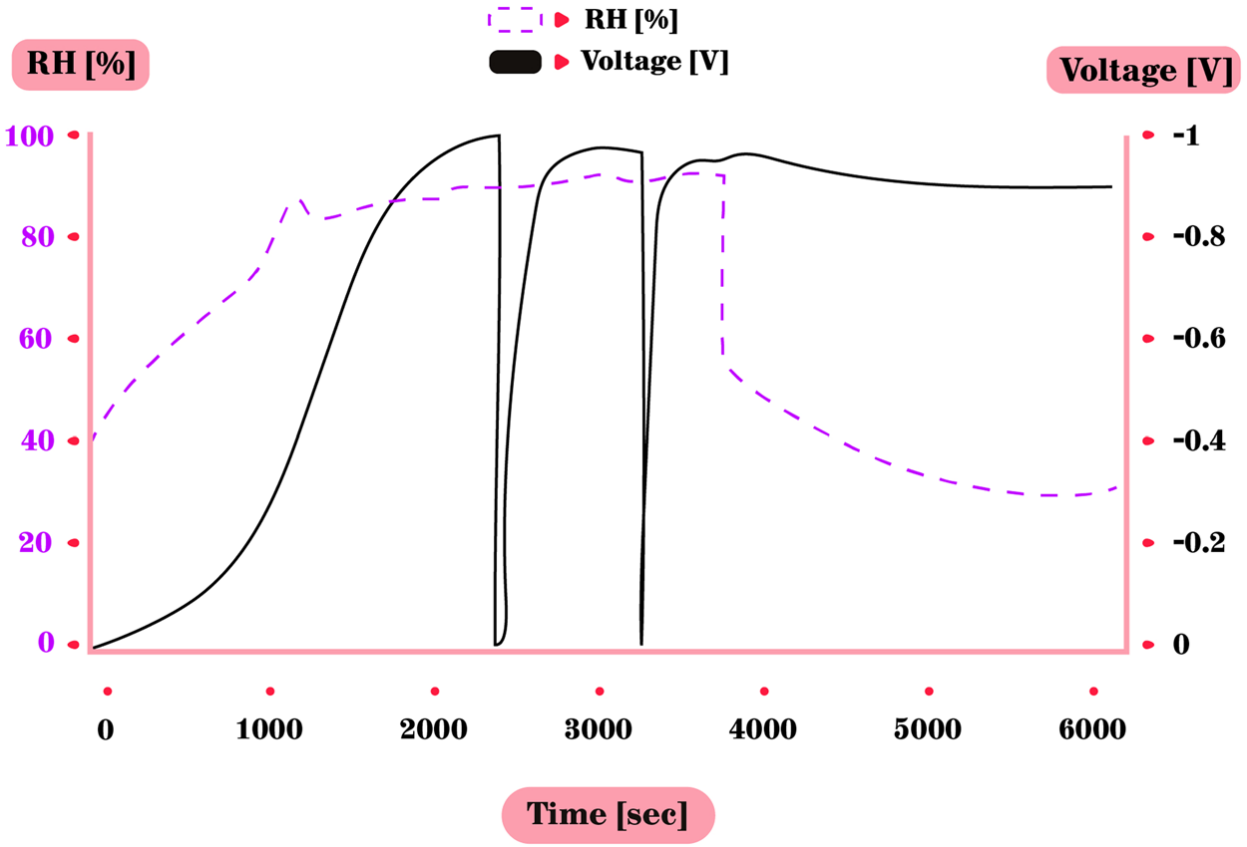
Voltage accumulation of an asymmetrical capacitor made of zinc and SS316LS plates. With every cycle of grounding-reconnecting the voltage recovered immediately. Accumulated voltage remained high for some time even after the RH was lowered.

Since different metals acquire different voltages between themselves and the outer grounded CPB, asymmetrical capacitors were built. This was done in order to examine the spontaneous voltage that can be obtained between two dissimilar metals (under high RH). The asymmetrical capacitors were built from two dissimilar metal plates, separated by a paper sheet (Fig. 5), rolled together into a spiral cylinder. Unlike the findings in a previous research^23^, in our experiments, a capacitor built from aluminum foil and a SS mesh did not reach a significant voltage. However, the type of SS was found to be crucial, hence it is possible that a different type of SS was used in the previous research, which led to their different results. While combinations of asymmetrical capacitors, made of SS-301 & SS-316LS or zinc & aluminum, generated a negligible voltage, asymmetrical capacitors made of SS-316LS and zinc or SS-316LS and SS mesh, reached up to 1 V spontaneously (Fig. 6). Connecting these capacitors in series generated higher voltages as expected, although the increase was not linear and the incremental voltage was smaller with every added capacitor.

### Outdoors experiments

As preparation for the outdoors experiment, we first checked whether the presence of the Faraday cage plays any role in terms of eliminating any outer interference which may affect our results. We also compared results when distilled or regular (tap) water were used as a humidity source and finally, unlike the N_2_ used in former experiments, we used regular air. Those changes from the original experiments were in order to try to mimic natural conditions to make sure our finding will be relevant to experiment carried outdoors as well. Duplicating experiments under those changes ended up with similar results.

Using zinc as the sample metal, voltage accumulation was measured outdoors, during day and night, under ambient conditions. The experiments were carried out in order to verify if the ambient humidity, together with the aerosols, as sea salt and urban pollutants in the atmosphere, can also produce similar electric potentials in a natural outdoors setting.

The first outdoor experiment (not shown) was carried out on the roof of the Geophysics Department at Tel-Aviv University, only 2.5 km away from the Mediterranean coast and only 0.5 km away from the Yad-Avner meteorological station operated by The Israel Ministry of Environment Protection (at a similar height). This experiment was taken under typical summer conditions. Here, beyond verifying that voltage does accumulate under ambient conditions as well as in the lab, we explored whether daytime westerly wind (Etesian winds together with the sea breeze^26,30^) coming from the Mediterranean will contribute to the voltage accumulation rate. Also, the proximity to the coast affects the concentration of salt in the air. The presence of salt in the air is formed by evaporation of sea spray, carried by the wind. Hence, the concentration decreases with distance from the sea^31^. However, changes in wind direction and the proximity to the coast did not affect the voltage, which did not exceed −0.8 V.

**Figure 7:**
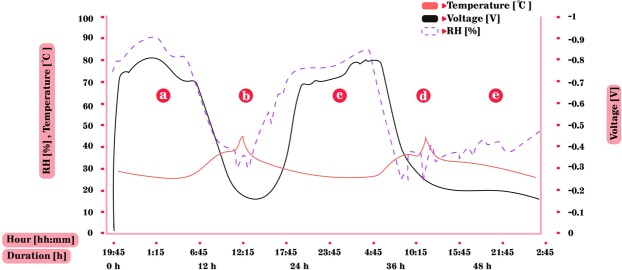
Outdoors 53 hours experiment – zinc and grounded CPB. (**a**) First night, RH is high; voltage accumulates to max of −0.8 V. (**b**) Daytime, temperatures are high, RH drops to very low levels and the voltage decreases gradually with the decrease of RH. (**c**) Second night, again with high RH and voltage accumulation followed. (**d**) Daytime, voltage decreases with increase in temperatures and decrease of RH. The weather is untypical with extremely dry conditions. (**e**) Third night with low RH due to the dry air. The temperatures decreased as in the former two nights but the RH increased to only 50% during the night hence, the voltage did not increase at all.

**Figure 8:**
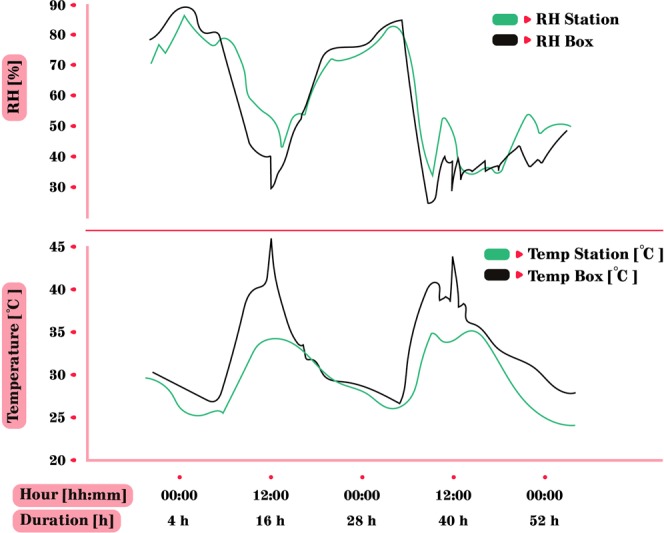
RH and temperature differences between the measurements at the IMS meteorological station and the direct measurements in the experiment box. Peaks in box temperature during noon were caused by direct sunlight on the box for a short while. The differences between the ambient RH and temperature readings of the meteorological station and the readings from the device measuring them directly mounted in the aluminum box are due to lower ventilation of the box. Those differences were more evident during daytime due to the heating of the sun.

The second outdoor experiment was a prolonged 53 hours experiment, exhibiting both typical summer conditions (with high RH) during the first two nights and extreme hot and dry conditions during the third night. This experiment was carried out in the suburbs of Tel-Aviv, downwind from the city center, at Kiryat Ono, 8 km away from the coast, with meteorological data taken from the Bet-Dagan station operated by The Israel Meteorological Service (IMS), 1 km southward, at a similar distance from the coast. The experiment started at 19:45 in the evening while the ambient RH was high (72%) and continued without being grounded for the entire duration of the experiment (Figs. 7 and 8). First, one can see the expected negative correlation between the RH (blue line) and the temperature (grey line) - while during noon hours when temperatures in the box exceeded 40 °C and the RH dropped to as low as 33%. At the same time, the conditions at the nearby meteorological station (IMS station, located at Bet-Dagan) were 32 °C and 52%, respectively. In the first two nights, as temperatures cooled, the RH reached 80% and higher. These high RH are typical to summer nights in this region^26^. The voltage accumulation between the zinc plate and a grounded CPB increased shortly after the increase in RH and reached the same maximum as in lab, −0.8 V. Following the sun rise, the temperatures in the box increased and the RH decreased, below 60%, at 6:30 local solar time (LST), and with it, the voltage decreased to a minimum point of approximately −0.15 V. An hour before sunset, at 17:30 LST, as temperatures decreased and RH increased – the voltage slowly recovered once again back to −0.8 V, as in the former night. However, under the extreme hot and dry third day, starting from the evening of 15.8.19, the RH during this night was lower than <50%. Accordingly, the voltage did not recover as before and remained low, as expected. This indicates the crucial effect of an extreme hot and dry events^27^.

## Conclusions

The research has successfully showed voltage accumulation between two dissimilar metals under high RH conditions, starting from RH > 60%, in lab as well as under outdoor conditions. This is in agreement with previous studies^23^, who found it from RH of 50%. These finding were attained only for certain types of metals, while others are found totally indifferent to high RH. Some differences were evident between our findings and former experiments; the CPB metal in previous experiments was the most successful one in terms of charge accumulation, although in our experiments it gained a very small voltage. Also, the capacitor built from SS and aluminum in our experiment reached lower voltage than the one described in the former experiment. However, since some of the metals participating in the experiments are alloys, it is most likely the metals we used are different from the ones used in previous experiments. Further investigation of different types of metals and alloys revealed that zinc and certain types of SS that were not mentioned in previous studies, are the most reliable metals to work with for voltage accumulation, among the metals tested. In most cases, the voltage generated between the tested metal and the grounded CPB was negative. It is only in two cases that we got positive voltage – in the case of tungsten and in a combination of SS wool with copper wire as backbone.

Building asymmetrical capacitors, made of two dissimilar metals, reached 1 V spontaneously under high RH. While the total voltage of capacitors connected in series is expected to be the sum of all the capacitors’ voltage, this was not the case in our experiment, as the incremental voltage from asymmetrical capacitors, connected in series, was not linear. Further research is needed in order to establish understanding of scaling up with multiple asymmetrical capacitors connected. In addition, due to the voltage similarities of some of the SS to those of zinc, we need to check whether the more successful SS are actually galvanized SS, i.e., zinc coated.

High RH, >60%, is found crucial for the voltage accumulation. However, it was found that under low temperatures, with similar RH, indicating lower AH, the accumulation was delayed. We have showed, for the first time, that voltage accumulation is also evident under ambient outdoor conditions, starting also from RH of 60%, and reaching the same voltage as in the controlled lab experiments.

The outdoor experiments, carried out in natural ambient air, also confirmed the ability of isolated zinc to acquire charge in an uncontrolled environment, reaching the same and even higher voltages outdoors, as in lab. A prolong experiment with changed conditions of RH and temperature, showed the importance of high RH as a facilitator of this phenomenon.

Future applications of this voltage accumulation could be used as a source for energy, especially in regions with persistent high RH conditions, either during nighttime or during both night and daytime. These conditions fit best tropical regions with high temperatures and RH (and high AH), as well as coastal regions even in dry areas, during nighttime.

## Supplementary information

Supplementary information.

## Data Availability

The data set of this work can be found at 10.5281/zenodo.3592791.
