# Supplementary material for: On the Spontaneous Build-Up of Voltage between Dissimilar Metals Under High Relative Humidity Conditions
Source: Sci Rep. 2020 May 6;10:7642. doi: 10.1038/s41598-020-64409-2 (PMC7203220; doi:10.1038/s41598-020-64409-2)
Supplement: Supplementary file 1 — Supplementary information. [file 41598_2020_64409_MOESM1_ESM.docx]

***Scientific Reports Journal***

Supporting Information for

**On the Spontaneous Build-Up of Voltage between Dissimilar Metals Under High Relative Humidity Conditions**

J. Y. Lax^1,*^, C. Price^1^ and H. Saaroni^1^

^1^Porter School of the Environment and Earth Sciences, Faculty of Exact Sciences, Tel Aviv University, Tel Aviv, Israel.

^*^Corresponding author: Judi Lax (yehuditlax@mail.tau.ac.il)

**Contents of this file**

Table S1

| Metal Type | Metal Symbol | Shape | Length | Width | Thickness | Diameter |
| --- | --- | --- | --- | --- | --- | --- |
| Zinc | Zn | Foil | 200 mm | 15 mm | 0.1 mm |  |
| Zinc | Zn | Cylinder | 230 mm | - | - | 26 mm |
| Zinc | Zn | Plate | 175 mm | 24 mm | 1 mm |  |
| Stainless Steel | SS 316 LVM | Rod | 100 mm |  |  | 1.5 mm |
| Stainless Steel | SS 316 LS | Foil | 200 mm | 100 mm | 0.12 mm |  |
| Stainless Steel | SS 316 L | Rod | 100 mm |  |  | 1.8 mm |
| Stainless Steel | SS 316 | Rod | 100 mm |  |  | 1.6 mm |
| Stainless Steel | SS 316 | Cuboid | 120 mm | 14.5 mm | 4.7 mm |  |
| Stainless Steel | SS 304 L | Rod | 100 mm |  |  | 0.4 mm |
| Stainless Steel | SS 304 L | Cuboid | 125 mm | 20 mm | 4.2 mm |  |
| Stainless Steel | SS 304 | Cuboid | 120 mm | 11 mm | 4.5 mm |  |
| Stainless Steel | SS 303 | Cuboid | 55 mm | 20 mm | 11 mm |  |
| Stainless Steel & Copper | SS & Cu | SS wool  with Cu wire  as backbone | 131 mm |  |  | 15 mm |
| Stainless Steel | SS | SS mesh | 70 mm | 25 mm |  |  |
| Stainless Steel | SS | SS mesh | 70 mm | 50 mm |  |  |
| Stainless Steel | SS | SS mesh | 70 mm | 100 mm |  |  |
| Stainless Steel | SS | Cable wire rope | 160 mm |  |  | 4 mm |
| Stainless Steel | SS 301 | Foil | 200 mm | 100 mm | 0.01 mm |  |
| Nichrome | NiCr | Wire | 200 mm |  |  | 0.4 mm |
| Aluminum | Al | Plate | 150 mm | 25 mm | 0.81 mm |  |
| Aluminum | Al | Cylinder shaped foil | 130 mm |  |  | 8 mm |
| Tin | Sn | Rod | 200 mm |  |  | 0.7 mm |
| Copper | Cu | Wire | 145 mm |  |  | 2.2 mm |
| Copper | Cu | Rod | 200 mm |  |  | 6 mm |
| Copper | Cu | Wool | 132 mm |  |  | 15 mm |
| Nickel | Ni | Rod | 200 mm |  |  | 8 mm |
| Chrome plated brass | CPB | Cylinder | 200 mm |  |  | 30 mm D |
| Chrome plated brass | CPB | Cylinder (outer) | 200 mm |  |  | 38 mm D |
| Silver | Ag | Plate | 50 mm | 25 mm | 1 mm |  |
| Molybdenum | Mo | Rod | 100 mm |  |  | 4 mm |
| Molybdenum | Mo | Rod | 200 mm |  |  | 4 mm |
| Tungsten | W | Rod | 250 mm |  |  | 3.2 mm |
| Brass | Brass | Plate | 200 mm |  |  | 30 mm |
| Brass | Brass | Cylinder | 89 mm |  |  | 30 mm |
| Indium | In | Wire  wrapped around a plastic rod | 55 mm |  |  | 6 mm |

**Table S1.** List of materials and dimensions used in the experiments
